# Exposure Assessment to Mycotoxins in a Portuguese Fresh Bread Dough Company by Using a Multi-Biomarker Approach

**DOI:** 10.3390/toxins10090342

**Published:** 2018-08-23

**Authors:** Susana Viegas, Ricardo Assunção, Carla Nunes, Bernd Osteresch, Magdalena Twarużek, Robert Kosicki, Jan Grajewski, Carla Martins, Paula Alvito, Ana Almeida, Carla Viegas

**Affiliations:** 1 H&TRC-Health & Technology Research Center, ESTeSL-Escola Superior de Tecnologia da Saúde, Instituto Politécnico de Lisboa, 1990-096 Lisboa, Portugal; ana.almeida@estesl.ipl.pt (A.A.); carla.viegas@estesl.ipl.pt (C.V.); 2 Centro de Investigação em Saúde Pública, Escola Nacional de Saúde Pública, Universidade NOVA de Lisboa, 1600-560 Lisboa, Portugal; cnunes@ensp.unl.pt; 3 Food and Nutrition Department, National Institute of Health Doutor Ricardo Jorge, I.P. (INSA), Av. Padre Cruz, 1649-016 Lisbon, Portugal; ricardo.assuncao@insa.min-saude.pt (R.A.); carla.martins@insa.min-saude.pt (C.M.); Paula.Alvito@insa.min-saude.pt (P.A.); 4 Centre for Environmental and Marine Studies (CESAM), University of Aveiro, Campus de Santiago, 3810-193 Aveiro, Portugal; 5 NOVA National School of Public Health, Universidade NOVA de Lisboa, 1600-560 Lisboa, Portugal; 6 Group of Prof. Humpf, Institute of Food Chemistry, Westfälische Wilhelms-Universität Münster Corrensstraße 45, 48149 Münster, Germany; osteresch@uni-muenster.de; 7 Department of Physiology and Toxicology, Institute of Experimental Biology, Faculty of Natural Sciences, Kazimierz Wielki University, 85-064 Bydgoszcz, Poland; twarmag@ukw.edu.pl (M.T.); robkos@ukw.edu.pl (R.K.); jangra@ukw.edu.pl (J.G.)

**Keywords:** fungi metabolites, biomonitoring, occupational exposure

## Abstract

Mycotoxins are toxic mold metabolites that can persist in environment long after the fungi species responsible for their production disappear. Critical workplace for mycotoxins presence has already been studied and nowadays it is possible to recognize that exposure to mycotoxins through inhalation occurs due to their presence in dust. This study aimed to assess occupational co-exposure to multiple mycotoxins in a fresh bread dough company, an occupational setting not studied until now. Occupational exposure assessment to mycotoxins was done using a LC-MS/MS urinary multi-biomarker approach. Twenty-one workers and nineteen individuals that were used as controls participated in the study. Workers/controls (spot-urine) and environment (settled dust) samples were collected and analyzed. Concerning workers group, DON-GlcA, and OTA were the most prevalent biomarkers (>LOD), 66% and 90.5%, respectively. In the control group, OTA was also one of the most detected (68%) followed by CIT (58%) and DON-GlcA (58%). DON was the mycotoxin measured in high amounts in the settled dust sample (58.2 ng/g). Both workers and controls are exposed to several mycotoxins simultaneously. The workers group, due to their high contact with flour dust, revealed a higher exposure to DON. Considering these results, risk management measures must be applied including specific and adequate health surveillance programs in order to avoid exposure and consequently the associated health consequences.

## 1. Introduction

Mycotoxins are well known active mold metabolites produced by specific fungal genera, primarily Aspergillus, Penicillium, Alternaria, and Fusarium genus [1,2]. They are small and quite stable molecules with low molecular mass in general. Nowadays, more than 300 secondary metabolites have been identified and it is expected that this number will increase in the near future. Among these metabolites, only about 30 have been studied aiming at highlighting their toxic proprieties [3].

Mycotoxins can persist in the environment even in the absence of visible mold since they resist adverse abiotic factors such as high or low temperatures and can be present in the environment long after the death; the disintegration of the fungi species is also responsible for their production [4,5,6]. They are also extremely difficult to eliminate from food even after the cooking process [7]. A specific fungal species can produce several different mycotoxins and production of mycotoxins occurs often only under certain environmental conditions. Moreover, mycotoxins are profoundly dependent on climate, plant, and storage-associated problems, and influenced by non-infectious factors (e.g., bioavailability of (micro) nutrients, insect damage, and other pests attack), that are also driven by climatic conditions. The increased risk of European countries with temperate climates to have higher exposure to fungi and mycotoxins due to climate change has already been identified by some authors [8,9], and this aspect must also be considered when predicting potential exposure to mycotoxins in specific exposure scenarios.

Critical workplaces for fungal contamination, and consequently for the presence of mycotoxins, that have already been studied are the feed/food industry, due to raw material processed or stored [10,11,12]; animal production due to feed and animal density [13,14,15,16]; slaughterhouses [17] due to the contact with contaminated animals; waste industry due to permanent availability of nutrients [18,19,20,21,22,23]; specific food processing plants with matrices easily contaminated by fungi, such as coffee [24], onions [25], and others such as sugar, grain products, spices, nuts, and corn [26,27,28].

Mycotoxins are commonly present in airborne dust [29,30] and in spores or fragments of microbial growth and both can act as mycotoxin carriers to the worker’s respiratory system [31]. Therefore, exposure in occupational settings occurs essentially by inhalation, particularly in the form of airborne dust [17,26,27,32,33,34]. Exposure can happen during tasks involving high contact with organic dust such as storing, loading, handling, or milling contaminated materials (grain, waste, and feed) and others such as handling of animals in animal production settings.

Human biomonitoring is a key role for the establishment of human exposure to mycotoxins [14,16,35,36,37,38]. Usually, studies consider food intake as the main route of exposure, based on estimated dietary intakes considering biomarker concentrations in urine and excretion rates. These approaches allow identifying exposure levels of the population below the tolerable daily intake (TDI) values set for certain mycotoxins. Biomonitoring covers, not only mycotoxin intake from all dietary sources, but also exposure by other exposure routes, such as inhalation of mycotoxins at the workplace [39]. Occupational exposure to mycotoxins is characterized as complex, since it is also associated to co-exposure to several mycotoxins by different exposure routes. Biomonitoring gives an answer to this specific need since there are already several analytical methods available that cover several mycotoxins simultaneously in different biological samples [37,38,40,41,42]. A few initial studies have been performed with the use of these important analytical methods to study occupational exposures to mycotoxins confirming that exposure is characterized by being multiple and occurring probably by different routes [39,43]. Whether workplace-related exposures could represent a significant mycotoxin exposure route compared to mycotoxin dietary intake through ingestion of contaminated food is a critical issue. As suggested by Föllmann et al. (2016), this should be investigated through comparison of results from workers and from non-occupationally exposed individuals (controls).

A recent study intending to review the levels of mycotoxins reported in food and feed products commercialized in Portugal showed that bread and other bakery products can be contaminated, although with low concentrations, by several mycotoxins, namely ochratoxin A (OTA), fumonisins from series B (FBs), zearalenone (ZEN), and trichothecenes such as deoxynivalenol (DON) [44]. Although mycotoxin concentration may be low, the raw materials used for the production of bakery products are handled by workers in high amounts and this can result in an elevated airborne mycotoxin concentration at the workplaces at a specific moment that endures depending on how the tasks are developed [27]. Another important feature in this occupational setting is the fact that exposure to organic dust is commonly observed [22,45,46] making this a perfect scenario for exposure by inhalation.

Considering the information above and the lack of studies on occupational exposure to mycotoxins in this particular setting, this study was developed aiming to assess co-exposure to multiple mycotoxins in a fresh bread dough company. Additionally, this study also aimed to understand what contamination sources are present in this setting that can influence exposure, and consequently, assess the associated risk.

## 2. Results

After task observation in each workplace, workers with higher contact with the dough and raw materials were invited to participate in this study. Twenty-one workers from a total of twenty-three were enrolled in this study.

### 2.1. Biomonitoring

#### 2.1.1. Participant Characteristics

The workers’ group of this study was composed of employees of one fresh bread dough company. The workers performed various tasks that are directly related to the fresh dough production process. The individuals integrating the control group were working in offices without expected occupational exposure to mycotoxins. As presented in Table 1, the mean ages in control participants were similar to those of the workers. The control group mean age was 41.3 years, ranging from 32 to 54 years. The workers group mean age was 35.2 years, ranging from 22 to 64 years.

#### 2.1.2. Mycotoxin Biomarkers in Urine

Table 2 and Table 3 provide an overview of the results obtained in both groups. Concerning workers group, DON-GlcA and OTA were the most prevalent biomarkers (>LOD), 95% and 48% respectively. In the control group, OTA was also one of the most detected (68%) followed by CIT (58%) and DON-GlcA (58%). Table 4 summarizes the mycotoxins urinary concentrations (>LOQ) in urine samples from workers and controls corrected to the creatinine content of a sample expressed as µg/g Crea. In the workers’ group, DON-GlcA presented the highest values, with a mean of 34.87 µg/g Crea and ranging from 12.60 and 64.51 µg/g Crea. EnA, DH-CIT, 2′*R*-OTA, and AFM_1_ were also detected in urine samples. AFM_1_ presented results above LOQ, with a mean of 4.21 µg/g Crea and ranging from 3.40 and 5.01 µg/g Crea. Regarding the control group, only one urine sample presented quantifiable values of CIT (24.2 ng/mL).

From the data set (Table 3 and Table 4), it is readily apparent that DON-GlcA was the most prominent biomarker found in both groups but at the highest levels in the samples from the workers’ group. AFM_1_ showed lower concentrations compared to DON-GlcA but also only measured in the workers group. OTA was detected in both groups showing that 58% (23/40) of all the individuals enrolled in the study were exposed. CIT was measured in only one sample from the control group.

### 2.2. Settled Dust

Environment samples (settled dust) were analyzed for the presence of thirty-six mycotoxins and their metabolites. The mycotoxins and metabolites detected are the ones reported in Table 5, namely: Deoxynivalenol-3-glucoside (D3G), DON, ZEN, monoacetoxyscirpenol (MAS), OTA, and mycophenolic acid (MPA). DON was clearly the most highly concentrated mycotoxin in the settled dust sample.

It was observed that, in overall the production stages, dust was in the air, particularly nearby the mixers and in specific parts of the machines that perform the cutting and the shaping of the dough. However, no respiratory protection devices were used by the workers involved in the process.

### 2.3. Exposure Assessment and Risk Characterization—DON Case

Considering DON was detected in a high concentration in the results, a more detailed analysis was performed. Considering the considerable DON-GlcA quantified results (>LOQ) for a significant percentage of workers (43%), special attention should be dedicated to the exposure to DON for these individuals. Table 6 presents the DON estimated exposure of fresh bread dough workers and control group derived through urinary levels of DON-GlcA. The highest DON estimated daily exposure was 1.04 µg/kg b.w./day which corresponds to a worker that is involved in the operation and manual supply of the dough mixers. Considering the established PMTDI for DON (1 µg/kg b.w./day), a percentage value considering the estimated exposure was calculated (Table 6). Two workers revealed estimated daily exposure values above the PMTDI for DON, exceeding 2% and 4% the established PMTDI. Additional workers revealed exposure values close (>80%) to the PMTDI. Despite some uncertainties associated to these results, it should not be ignored that these exposure results could contribute to the development of toxic health effects.

Comparing the DON exposure means between the two groups (workers and control), a significant statistical difference (*p* = 0.026) was verified, supporting the hypothesis that besides ingestion an additional route of exposure to DON can be considered for the workers group.

**Figure 1:**
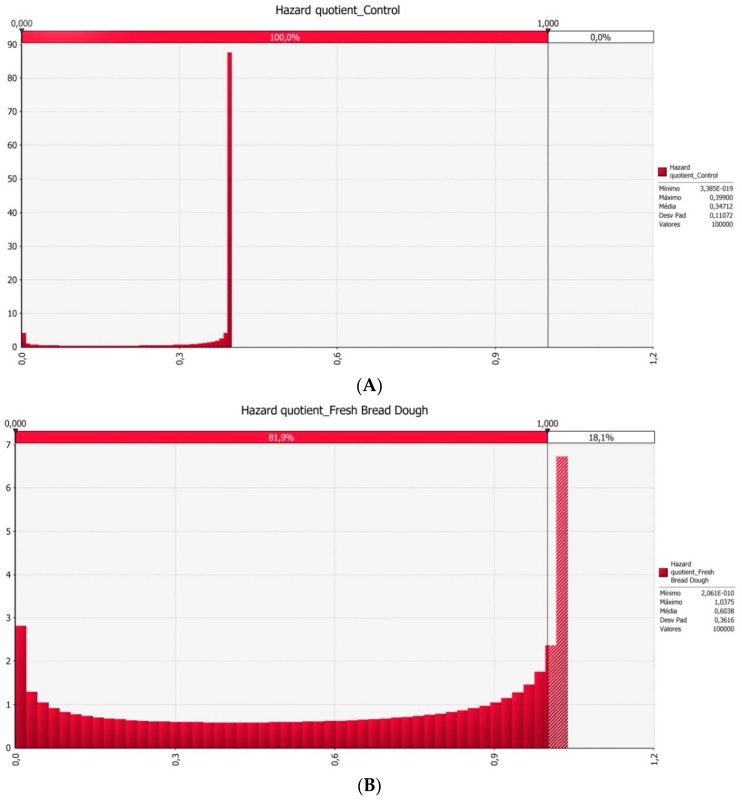
Distribution of hazard quotient (HQ) using @Risk for control individuals (**A**) and fresh bread dough workers (**B**). Individual exposure values were fitted to a best fit probabilistic distribution and HQ were calculated. A vertical line scoring HQ = 1 was added, representing the cut-off level. HQ above 1 suggests a non-tolerable exposure level.

Regarding the data variability and uncertainty, a stochastic approach for the risk characterization was applied. Figure 1 presented the distribution of HQ to control individuals (A) and fresh bread dough workers (B). Data analysis shows that about 18.1% of the workers could exceed DON’s PMTDI and consequently potential health consequences could occur.

## 3. Discussion

As far as the authors know this is the first study developed with the purpose of assessing occupational exposure to mycotoxins in a fresh bread dough company. Previously, and related to this setting, only grain farms were studied regarding occupational exposure to mycotoxins [10,34,47,48,49,50]. Additionally, only one recent paper use biomonitoring tools in the grain industry to assess occupational exposure to mycotoxins [39]. Moreover, in all the previously published papers, DON exposure was frequently reported and DON was the mycotoxin with the higher concentrations found in the analyzed biological samples. These previously reported results explain the settled dust sample result since it is essentially composed of raw materials based on different grains, depending on the bread being produced. In the present study, DON-GlcA was the mycotoxin urinary biomarker more detected, being a matter of concern since DON is associated with delayed growth as well as immunotoxic and hepatotoxic effects [51,52].

Previously, an experimental study of deoxynivalenol biomarkers in urine supported by EFSA and developed by Brera et al. [53] and including three European countries allowed us to understand from what kind of food commodities exposure was resulting. In Italy, intakes of pasta and pasta-like products were significantly associated with higher levels of total DON after correction for creatinine. In Norway, intakes of breakfast cereals and snacks, and bread and bread-like foods were significantly associated with a higher level of total DON adjusted for creatinine. In the UK, biscuit intakes were significantly associated with a higher level of the toxin. Therefore, this also demonstrates that the contamination is probably coming from the crops, continues in the grain farms where the grains are processed and storage to produce various products namely feed and flours industry. Probably, this is the reason for the difference found between workers and controls urine samples regarding the DON biomarker (DON-GlcA), where all the results higher than the LOQ were found in the workers group. Therefore, for this particular mycotoxin, workplace exposure adds significantly to the exposure resulting from ingestion of mycotoxin-contaminated food. Even if the mycotoxin content is low and below legal reference concentrations in the raw materials used for bakery products production such as grains and flours, we should consider that these materials are handled in high amounts and in a daily-basis by workers and this probably results in an elevated airborne mycotoxin concentration in the workplaces at specific moments [27]. Due to careful control of the time/temperature combination during bread baking, a minimization of the DON level in the final bread occurs [54]. However, this stage does not occur in the company enrolled in the study and, consequently, this could explain the difference between workers and controls.

DON does not require metabolic activation to exert its biological effects. Low-level trichothecene exposure in animal models has been shown to modulate the expression of several cytokines and chemokines that are key regulators of immune function [55]. Exposure to DON causes the upregulation of the mRNAs responsible for the production of cytokines, chemokines, and other immune-related proteins and can also induce gene transcription. In addition, DON modulates numerous physiological processes controlled by mitogen-activated protein kinases (MAPKs). These include processes controlling cell growth, differentiation, and apoptosis, which are all crucial for signal transduction in the immune response [55]. Thus, in addition to altered cytokine expression, alterations in MAPK expression are likely to also contribute to the immune dysregulation and toxicity of DON and other trichothecenes. Also associated with MAPK activation by DON is the activation of processes leading to the ribotoxic stress response, which is induced by other translational inhibitors that, like DON, which bind to or damage a specific region at the 3′ end of the 28S rRNA. The ribosome plays a key role in the ribotoxic stress response by serving as scaffolding for interactions between various MAPKs [55].

Previously, some epidemiological studies have been conducted in Norway concerning occupational exposure of male and female farmers to mycotoxins. Norwegian grains (wheat, oats, and barley) are affected mainly by Fusarium head blight, and various trichothecenes such as DON have been commonly reported [56,57]. A longitudinal survey of farmers over more than two decades suggested a relationship between grain farming and mid-pregnancy deliveries in the families of farmers, possibly linked to mycotoxins [58]. Small increased relative risks were observed in several cancers in female but not male farmers [58].

In the present study, in order to characterize the associated risk to the exposure level, an approach commonly used to derive exposure levels, based on excretion and conversion rate of mycotoxins was applied. Through this, it was estimated that 18.1% of workers could be at health risk. However, considering the verified differences in the exposure levels between the two groups included in the study (workers and control group) it could be anticipated that other routes of exposure, namely inhalation, but also swallowing after contact with the upper respiratory system, should be considered. The applied approach, despite including some uncertainty, allows us to characterize the potential associated risk and enables us to infer about the need for the implementation of risk management measures to reduce the associated health consequences.

Considering that exposure to DON is also occurring by inhalation, it is also important to highlight that exposure to mycotoxins by inhalation can be more relevant regarding health effects than exposure by ingestion [36]. Besides this, we are probably facing peak exposures occurring due to tasks developed in a way that promotes particle aerosolization. This is different from the exposure that happens due to food intake since in this case the exposure is characterized normally as chronic (low amounts in a constant manner). This particular difference can result in different toxicokinetics and, in some cases, imply different health effects from the ones already reported. Concerning the results obtained for the supplementary mycotoxins biomarkers found in workers and controls (AFM_1_, EnB, CIT, DH-CIT, OTA, and 2′*R*-OTA) the exposure sources and routes are probably different and, due to the low concentrations found, exposure is apparently occurring by food consumption. This is supported by the fact that the results are similar to the ones found in previous studies where exposure was related to food intake [37,59]. Regarding AFM_1_, that is the hydroxilated metabolite of aflatoxin B1 (AFB1) found in milk of animals exposed to AFB1, and was determined in both groups (workers and controls), it is important to mention that this can imply exposure to aflatoxin B_1_, a potent hepatocarcinogen because of its involvement in the etiology of human liver cancer and classification as Group 1 (human carcinogen) by the International Agency for Research on Cancer [60]. The carcinogenicity of AFM_1_ is considered as ten times lower than that of AFB_1_ and has been classified as ‘‘possibly carcinogenic to humans’’ (group 2B) by IARC [60].

OTA findings in both groups can easily be explained due to the common presence of this mycotoxin in several food commodities such as cereals, beer, coffee, wine, cocoa, dried fruits, meat, and spices since it is a stable compound that is not destroyed by common food processing [61,62,63,64]. OTA is produced by different Aspergillus and Penicillium species and is one of the most abundant food-contaminating mycotoxins.

Previous studies developed in the Portuguese population already reported OTA in biologic fluids [63,64] relating to the consumption of some food commodities. A study developed by Lino et al. (2008) compared the results obtained in the Portuguese population with the results from other European countries and it was possible to conclude that estimated daily intake values in the Portuguese populations are higher than other European populations [63]. This explains the fact of OTA being detected in almost all the urine samples (80%), despite below the LOQ. The same results tendency was found in a previous paper that aimed to study occupational exposure to mycotoxins in the waste management workers [43], where it was shown that besides AFB_1_ occupational exposure the results obtained for OTA and EnB were probably due to dietary intake. However, and in a more detailed manner, coffee seems a more relevant source of exposure to OTA in the group of controls since 2′*R*-OTA, an OTA degradation product formed only during coffee roasting and considered less toxic than its precursor [65], was detectable in four individuals of that group.

Regarding EnB results, a previous paper also reported this mycotoxin in waste management workers plasma [43] and, like in the present study, the results were very low and probably due to food intake [66]. This is probably due to the high prevalence of these mycotoxins in Portuguese wheat-based cereals [66,67]. Although it was detected in a small number of samples, its occurrence is of concern, since its cytotoxicity is comparable to that of DON [66,68]. Citrinin, a nephrotoxic mycotoxin produced by fungi of genera Penicillium, Aspergillus, and Monascus, is a common food contaminant and it is generally formed after harvest and occurs mainly in stored grains, but also in other plant products such as beans, fruits, fruit and vegetable juices, herbs and spices, and also in spoiled dairy products [69,70]. Dihydrocitrinone (DH-CIT) is the main metabolite of CIT in human urine [71]. In the same way, regarding the present study, exposure seems to result from the diet. An additional aspect that it is relevant to consider is the fact that both groups (workers and controls) are subject to a multi and simultaneous exposure to several mycotoxins resulting from different contexts and exposure routes. The use of a multi-biomarker approach allowed us to reinforce this reality once more. This is of particular relevance, and is challenging for the risk assessment process since we are dealing with, besides a simultaneous exposure to multiple mycotoxins, exposure resulting probably from different exposure routes and with different regimes and frequency (chronic vs. acute exposure). Previous works studying the occurrence in food or the exposure to multiple mycotoxins of Portuguese population groups also reflected this reality [72,73].

It is important to refer that besides the small size of the sample (workers and control group) and the fact that the diet of both groups was not controlled, the present study included others sources of uncertainty, namely (i) the calculations to derive the DON exposure levels were based on excretion ratio assumptions and inter-individual variations were not taken into account; (ii) only the DON-GlcA metabolite was considered, despite other metabolites could occur; (iii) considering the inexistence of toxicokinetic data concerning exposure through inhalation and the risk was assessed considering the available toxicokinetic data from food resources).

## 4. Conclusions

In summary, from this study there is some evidence that workers and controls are exposed to several mycotoxins simultaneously, and workers, due to their high contact with flour dusts, have higher exposure to DON. Therefore, this exposure is probably due to raw materials contamination and the manual handling of those materials. Being that it is difficult to eliminate contamination from raw materials, other risk management measures should be applied such as guaranteeing the closed and automatic supply of the raw materials to the production process, avoiding manual contact and, for specific and short duration tasks that can imply higher exposure to dusts (e.g., adjustment of the dough cutting machines), the workers should use respiratory protection equipment. Besides this, specific and adequate health surveillance programs should be defined to tackle also the exposure to mycotoxins. Additionally, further studies should be developed aiming to identify how predicted climate change can affect occupational exposure to mycotoxins in this and other settings. This is of particular relevance since cereal crop infection by fungi is particularly influenced by climacteric conditions.

## 5. Materials and Methods

This study was conducted in June 2017 in one fresh dough company in the scope of an enlarged exploratory study intending to characterize occupational exposure to particles, fungi, and mycotoxins in the Portuguese bread production setting.

### 5.1. Fresh Bread Dough Company Studied and Production Workflow

The fresh bread dough company is located in Portugal, Lisbon district. It has 60 workers distributed to several working shifts and two areas, namely production and logistics.

**Figure 2:**
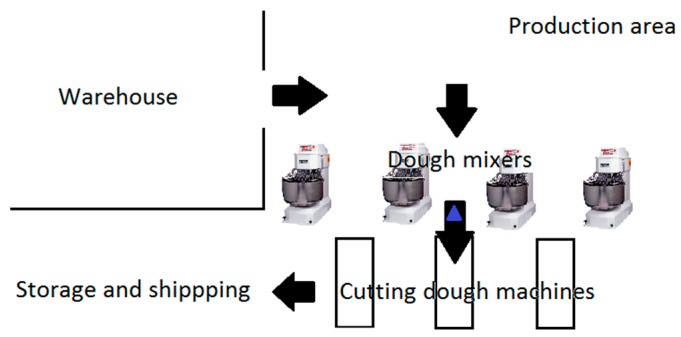
Description of the production area.

The production area, where this study was performed has got two separate areas: the raw materials warehouse and the production area. In the production area, four dough mixers are supplied by an automatic process but frequently some ingredients have to be manually handled by the workers (weighting and supplying machines). After the mixing process, the dough is distributed by three different production lines to cut the dough and give the desired shape. At the end of the line, the dough has the shape of the bread that it is being produced. During all process, there are workers responsible for supplying the line with the mixed dough, to adjust the machines function and to receive and pack the bread. At the end of the line the fresh dough will be finished by baking in a bakery shop after transportation (Figure 2).

### 5.2. Biomonitoring Approach

Occupational exposure assessment to mycotoxins was performed using a multi-biomarker approach since it allows a more precise and realistic exposure assessment over a broad range of different compounds. This approach is of particular importance as it allows recognizing and determining the presence of a multiple or rather simultaneous exposure to several mycotoxins. Only workers that developed their tasks in the production area for 7 h per day engaged in the biomonitoring (*n* = 21).

A control group (not exposed) was recruited (*n* = 19), besides workers, in order to investigate mycotoxin background levels for the Portuguese population and to evaluate and detect easier putative possible differences regarding exposure of the workers group. Therefore, the control group was composed of individuals who conducted administrative tasks in an educational institution without any type of activity known to involve occupational exposure to mycotoxins. In this study it is assumed that both groups (workers and controls) have similar diets and consequently it was hypothesized that the main difference on exposure to mycotoxins was the work activities.

This study was conducted in full accordance with the World Medical Association Declaration of Helsinki [74]. Written consents from the participants involved in this study were obtained. All participants were informed about the scope and aim of this study and signed a consent form.

Additionally, during a personal interview the participants answered a questionnaire to collect personal data, such as age, detailed current and previous occupational history, tasks performed in the two previous days prior urine collection, as well as activities outside the company (such as agriculture or animal production).

Workers spot urine samples were collected on the same day and between 11:00 a.m. and 4:00 p.m. in the medical center available in the company.

#### Urine Analysis

After collection, all urine samples were stored at 4 °C during transportation to the laboratory (<2 h) and then frozen at −20 °C until analysis (4–8 months). A LC-MS/MS urinary multi-biomarker approach was used to analyze the samples following a dilute-and-shoot sample preparation after centrifugation of thawed samples.

For this, samples were taken out of the freezer, reached room temperature and were centrifuged at 15,000× *g* for 10 min. In a 96-well plate, an aliquot of 11.1 µL was mixed with 100 µL of the LC-eluent with starting conditions of the gradient (95/5/0.1, water/acetonitrile/formic acid, *v/v/v*) in order to prepare a dilution of 1:9, which was ready for injection. All samples were analyzed in duplicate. Investigated compounds were the aflatoxins B1/2/G1/2/M1 and the Alternaria toxins alternariol, alternariol–monomethyl ether, and altenuene. Furthermore, citrinin and its urinary metabolite dihydrocitrinone were analyzed, along with ochratoxin A, 2′*R* ochratoxin A, 10 hydroxyochratoxin A, and ochratoxin α. Also, the trichothecenes deoxynivalenol, deoxynivalenol-3-glucuronic acid conjugate, together with T-2 toxin, HT-2 toxin, and HT-2-4-glucuronic acid conjugate were part of the investigation. In addition, the fumonisins B1/2 and zearalenone, zearalanone, α- and β zearalenol as well as zearalenol-14-glucuronic acid conjugate were included. Lastly, the enniatins A/A1/B/B1 and their structurally related beauvericin were incorporated into this method. In short, for analysis a 1260 Infinity LC system (Agilent, Santa Clara, CA, USA) coupled to a QTRAP 6500 mass spectrometer (SCIEX, Santa Clara, CA, USA) was used. Chromatographic separation of an injection volume of 30 µL was realized by using a C18 Pyramid column (Macherey-Nagel, Bethlehem, PA, USA) with a gradient of water and acetonitrile, both with addition of 0.1% formic acid, an oven temperature at 45 °C, and a flow of 600 µL/min. The mass spectrometer was operated with Analyst 1.6.2 in both polarities with electrospray ionization at +5500 V or 4500 V, a source temperature at 500 °C, curtain gas at 40 psi, nebulizer gas at 45 psi, and heater gas at 55 psi. Detailed parameters can be found as described previously [37].

Creatinine was determined in workers spot urine samples to correct for differences in dilution between individuals and excretion rates. Determination of urinary creatinine was performed in an automatized equipment (Dimension RXL, Siemens^®^, Munich, Germany) with a spectrophotometric method based on Jaffe reaction. Results for mycotoxins urinary concentrations were expressed as µg mycotoxin/g creatinine.

### 5.3. Settled Dust

From the workplace environment a settled dust sample was collected into a sterilized bag. This sample was collected at the end of a shift and in the floor nearby the dough mixers (marked with a triangle in Figure 2). Since it was possible to collect only one sample, this sampling location was chosen because it represents all the contamination present in the raw materials used for the production of that work shift. Therefore, it shows the overall mycotoxins contamination coming from the raw materials.

After sampling, 4.4 g of the collected floor settled dust were weighted and extracted with 40 mL of distilled water for 20 min at 200 rpm, as previously described [75,76,77]. Samples were frozen at −20 °C until analysis.

#### 5.3.1. Chemicals

All mycotoxin standards (except for griseofulvin and mevinolin—purchased from Sigma-Aldrich, St. Louis, MO, USA) were bought in Romer Labs (Tulln, Austria). Ammonium acetate, acetic acid, acetonitrile (gradient grade), and methanol (LC-MS grade) were purchased from Merck (AG, Darmstadt, Germany). Deionized water was obtained using a Simplicity UV water purification system (Millipore, MA, USA).

#### 5.3.2. Analysis of Settled Dust

Aliquots from settled dust (0.25 g) were homogenized with 1.0 mL of acetonitrile (ACN): water (H_2_O): acetic acid (AcOH) (79:20:1, *v/v/v*) for 60 min. Raw extracts were diluted with the same amount of water, mixed, filtered, and injected into the LC-MS/MS system. Detection of mycotoxins was carried out using high performance liquid chromatography (HPLC) Nexera (Shimadzu, Tokyo, Japan) with a mass detector API 4000 (Sciex, Foster City, CA, USA). Mycotoxins were separated on a chromatographic column Gemini NXC18 (150 × 4.6 mm, 3 μm) (Phenomenex, Torrance, CA, USA); mobile phase (A: water + 5 mM ammonium acetate + 1% acetic acid, B: methanol + 5 mM ammonium acetate + 1% acetic acid) with a mobile phase flow rate of 0.75 mL/min and an injection volume of 7 μL. The mycotoxin concentration was calculated using external calibration. Several mycotoxins and their metabolites were assessed, namely patulin, nivalenol, deoxynivalenol-3-glucoside, deoxynivalenol, fusarenon-X, deepoxy-deoxynivalenol, α-zearalanol, β-zearalanol, β-zearalenol, α-zearalenol, zearalenone, T-2 toxin, T-2 tetraol, T-2 triol, neosolaniol, 15-acetyldeoxynivalenol, 3-acetyldeoxynivalenol, monoacetoxyscirpenol, diacetoxyscirpenol, aflatoxin M_1_, aflatoxin B_1_, aflatoxin B_2_, aflatoxin G_1_, aflatoxin G_2_, fumonisin B_1_, fumonisin B_2_, fumonisin B_3_, roquefortine C, griseofulvin, HT-2 toxin, ochratoxin A, ochratoxin B, mycophenolic acid, as well as mevinolin. Table 7 summarizes the parameters of the optimized multiple reaction monitoring (MRM) transitions and ion source settings.

The limits of detection (LOD) and quantification (LOQ) obtained for each mycotoxin with this analytical method used are referred in Table 8. The LOD and LOQ values were calculated from mycotoxins standards spiked into a blank settled dust extract and into a pure solvent. The LOD and LOQ values were calculated based on signal-to-noise (S/N) ratios of 3:1 and 10:1, respectively, using the Analyst 1.6 software, version 1.6.2 (Sciex, ON, Canada, 2012).

### 5.4. Statistical Analysis

Statistical analysis was performed using IBM^®^ SPSS Statistics 20 software (IBM, Armonk, NY, USA). Descriptive statistics are presented as means (±standard deviation) and range (minimum and maximum). Differences in means of mycotoxin concentrations between the control group and workers were evaluated through independent sample *t*-test. The level of *p* ≤ 0.05 was considered statistically significant.

### 5.5. DON Exposure Assessment and Risk Characterization

The estimation of individual exposure to DON (µg/kg b.w./day) was performed based on the determined urinary levels of DON-GlcA, according to the Equation (1):

(1) $$Estimated\;exposure\;\left(\text{µ}g/\mathrm{kgb}.w./\mathrm{day}\;\right)=\frac{\left(DON-GlcA\times 0.63\right)\times V}{ER\times b.w.\times 1000}$$

With *DON − GlcA* = urinary concentration (ng/mL), considering a correction factor (0.63) for calculation of DON-equivalents; *V* = daily urine production (assumed as 24 mL/kg b.w./day); *ER* = urinary excretion ratio of DON (72%); *b.w.* = individual body weight (kg). For results of DON-GlcA urinary concentration not detected (below the LOD), half of LOD value was assumed. For DON-GlcA urinary concentration below the LOQ, half of LOQ value was assumed. The substitution of non-detects with other values is widely used in food risk assessment and since leads to conservative estimates for exposure assessment calculations it was also used in this case [78]. This approach is also in agreement with common practice in occupational exposure measurements.

Considering the unavailability of Human Biomonitoring (HBM)-related guidance values (HBM values or Biomonitoring equivalents as reviewed by Choi et al., 2015 [79] for urinary biomarkers of DON for risk characterization purposes, the estimated exposure values were compared to the established TDI (for DON, PMTDI = 1 µg/kg b.w./day according to JECFA, 2011). Although the main expected workers’ exposure route was inhalation, the exceeding TDI values can imply possible health risk for the workers. Following the traditional approach for the risk assessment, risk characterization step was executed through the calculation of the hazard quotient (HQ), as a ratio between exposure and the established PMTDI for DON. As generally accepted, if HQ < 1 indicates a tolerable exposure level and a ratio of HQ > 1 indicates a non-tolerable exposure level [48].

In order to represent the variability of exposure levels, a probabilistic approach was applied, using the software @Risk^®^ for Microsoft Excel version 7 (Palisade Corporation, Ithaca, NY, USA, 2016). The best fit function of @Risk software was applied in order to select the best fitted probabilistic distribution for the individual exposure levels, based on lowest Akaike’s Information Criterion (AIC). Monte Carlo simulations were performed considering 100,000 iterations. The percentage of workers that exceed the HQ value of 1 was estimated, revealing the percentage of workers population that should be under health risk.

## Figures and Tables

**Table 1:** Age and gender distribution of workers and control groups, including the number of years of activity.

Groups	Female	Male	Age (Mean; SD)	Years of Activity (Mean; SD)
Workers (*n* = 21)	9	12	35.2; 11.1	5.4; 5.1
Controls (*n* = 19)	6	12	41.3; 5	-

**Table 2:** Mycotoxins biomarkers detected in urine samples from workers and controls.

Groups	DON-GlcA	AFM_1_	EnB	CIT	DH-CIT	OTA	*2′R*-OTA
LOD (µg/L)	1.24	0.11	0.006	0.61	0.115	0.011	0.036
LOQ (µg/L)	4.14	0.38	0.020	2.00	0.383	0.036	0.120
**Workers (*n* = 21)**							
>LOQ (*n*, %)	3, 14%	-	-	-	-	-	-
LOD-LOQ (*n*, %)	17, 81%	3, 14%	3, 14%	6, 29%	3, 14%	10, 48%	2, 10%
<LOD (*n*, %)	1, 5%	18, 86%	18, 86%	17, 71%	18, 86%	11, 52%	19, 90%
**Controls (*n* = 19)**							
>LOQ (*n*, %)	-	-	-	1, 5%	-	-	-
LOD-LOQ (*n*, %)	11, 58%	1, 5%	2, 11%	10, 53%	2, 11%	13, 68%	4, 21%
<LOD (*n*, %)	8, 42%	18, 95%	17, 89%	8, 42%	17, 89%	6, 32%	15, 79%

**Table 3:** Mycotoxins biomarkers detected in urine samples from workers and controls.

Groups	DON-GlcA LOD = 1.24 LOQ = 4.14	AFM_1_ LOD = 0.11 LOQ = 0.38	EnB LOD = 0.0059 LOQ = 0.020	CIT LOD = 0.61 LOQ = 2	DH-CIT LOD = 0.115 LOQ = 0.383	OTA LOD = 0.011 LOQ = 0.036	*2′R*-OTA LOD = 0.036 LOQ = 0.12
Workers (*n* = 21)	>LOQ = 9 (43%) <LOQ = 5 (24%)	>LOQ = 2 (10%)				<LOQ = 19 (90.5%)	
Controls (*n* = 19)	<LOQ = 11 (58%)	<LOQ = 1 (5%)	<LOQ = 2 (11%)	>LOQ = 1 (5%) <LOQ = 10 (53%)	<LOQ = 10 (53%)	<LOQ = 13 (68%)	<LOQ = 4 (21%)

**Table 4:** Mycotoxins biomarkers levels (>LOQ) in urine samples from workers (µg/g Crea) and controls (ng/g).

Groups	DON-GlcA	AFM_1_	CIT
**Workers (µg/g Crea)**			
Range	12.60–64.51	3.40–5.01	
Mean	34.87	4.21	
SD	17.45	1.14	
**Controls (ng/mL)**			
Single value			24.2 *

* not corrected for creatinine concentration.

**Table 5:** Mycotoxins detected in the settled dust sample (ng/g).

Mycotoxins	Concentration
D3G	<LOQ
DON	58.2
ZEN	<LOQ
MAS	0.54
OTA	<LOQ
MA	0.84

**Table 6:** Deoxynivalenol estimated exposure of fresh bread dough workers and control group based on urinary levels of DON-GlcA. The percentage of the tolerable daily intake (TDI) for DON correspondent to the estimated exposure was presented. For results of DON-GlcA urinary concentration below the LOD, or below the LOQ, half of the LOD value and half of the LOQ value were assumed, respectively.

Participants	DON Estimated Daily Exposure (µg/kg b.w./day)	Percentage of TDI
Control 1	0.12	12
Control 2	0.12	12
Control 3	0.12	12
Control 4	0.40	40
Control 5	0.40	40
Control 6	0.40	40
Control 7	0.40	40
Control 8	0.12	12
Control 9	0.40	40
Control 10	0.12	12
Control 11	0.12	12
Control 12	0.40	40
Control 13	0.40	40
Control 14	0.12	12
Control 15	0.40	40
Control 16	0.12	12
Control 17	0.40	40
Control 18	0.40	40
Control 19	0.40	40
Worker 1	0.71	71
Worker 2	0.12	12
Worker 3	0.12	12
Worker 4	0.97	97
Worker 5	0.40	40
Worker 6	0.54	54
Worker 7	0.43	43
Worker 8	0.40	40
Worker 9	1.04	104
Worker 10	0.12	12
Worker 11	0.12	12
Worker 12	0.40	40
Worker 13	0.81	81
Worker 14	0.12	12
Worker 15	0.12	12
Worker 16	0.12	12
Worker 17	0.71	71
Worker 18	0.40	40
Worker 19	0.40	40
Worker 20	0.65	65
Worker 21	1.02	102
Tolerable daily intake of DON = 1 µg/kg b.w./day		

**Table 7:** Optimized ESI-MS/MS conditions for analytical method.

Compound	Precursor Ion (*m/z*)	Declustering Potential (V)	Product ions (*m/z*) *	Collision Energy (V)	Cell Exit Potential (V)
Patulin	152.9	−55	108.9/81.0	−12/−16	−5/−3
Nivalenol	371.1	−60	281.0/59.0	−22/−38	−5/−1
Deoxynivalenol−3-Glucoside	517.2	−70	427.1/457.2	−28/−20	−9/−11
Deoxynivalenol	355.1	−55	265.0/59.0	−22/−38	−11/−10
Fusarenon X	413.1	−35	263.1/186.9	−22/−36	−10/−11
Deepoxy-deoxynivalenol	339.1	−50	249.2/59.0	−16/−34	−15/−10
α-Zearalanol	321.1	−110	277.0/303.1	−30/−30	−5/−7
β-Zearalanol	321.1	−110	277.0/303.1	−30/−30	−5/−7
β-Zearalenol	319.0	−110	159.8/173.9	−42/−36	−11/−11
α-Zearalenol	319.0	−110	159.8/173.9	−42/−36	−11/−11
Zearalanon	319.0	−105	204.8/161.0	−32/−38	−13/−9
Zearalenon	317.1	−110	131.0/174.9	−40/−34	−7/−11
T2 Tetraol	361.2	56	215.0/281.1	13/13	14/20
Neosolaniol	400.2	51	185.1/245.1	29/17	12/16
15-Acetyldeoxynivalenol	356.1	41	321.0/137.1	19/21	22/8
3-Acetyldeoxynivalenol	339.2	71	231.1/203.1	17/21	16/14
Monoacetoxyscirpenol	342.1	41	265.1/107.1	13/21	18/6
Diacetoxyscirpenol	384.2	51	307.0/247.0	17/21	20/16
Aflatoxin M1	329.0	71	273.1/259.0	33/33	18/18
Aflatoxin B1	313.1	106	285.1/128.1	33/93	20/8
Aflatoxin B2	315.1	106	287.1/259.0	37/41	20/18
Aflatoxin G1	329.1	96	242.9/200.1	37/57	16/14
Aflatoxin G2	331.0	76	189.0/245.1	59/45	13/12
Fumonisin B1	722.4	116	334.4/352.3	57/51	10/10
Fumonisin B2	706.4	121	336.4/318.3	51/55	10/10
Fumonisin B3	706.4	121	336.4/318.3	51/55	10/10
T2 Triol	400.2	36	215.2/233.1	17/13	14/16
Roquefortine C	390.2	81	193.0/322.0	39/29	12/22
Griseofulvin	353.1	71	165.0/215.1	29/29	10/14
T−2 toxin	484.2	56	215.0/305.1	29/21	14/8
HT−2 toxin	442.2	61	215.1/263.0	19/19	14/18
Ochratoxin A	404.1	61	239.0/358.1	33/21	16/10
Ochratoxin B	370.1	61	205.0/187.1	29/49	14/12
Mycophenolic acid	321.1	66	207.0/303.0	29/15	14/20
Mevinolin	405.3	81	285.1/199.1	17/17	20/12

Source temperature: 500 °C; curtain gas 20 psi; ion source gas 1 (sheath gas) 50 psi; ion source gas 2 (drying gas) 50 psi, ion spray voltage −4000 V (negative polarity) and 4500 V (positive polarity). * values are given in the order quantifier/qualifier.

**Table 8:** Estimated LODs and LOQs values in the blank settled dust matrix and in the standard solution.

Mycotoxins	LOD (Settled Dust Matrix) (ng/g)	LOQ (Settled Dust Matrix) (ng/g)	LOD (Standard Solution) (ng/mL)	LOQ (Standard Solution) (ng/mL)
Patulin	1.1	3.6	0.8	2.6
Nivalenol	4.5	14.9	3.6	11.9
Deoxynivalenol-3-glucoside	5.4	17.8	4.2	13.9
Deoxynivalenol	2.7	8.9	2.3	7.6
Fusarenon-X	4.8	15.8	4.2	13.9
Deepoxy-deoxynivalenol	4.2	13.9	3.3	10.9
α-Zearalanol	2.0	6.6	1.7	5.6
β-Zearalanol	0.9	3.0	0.9	3.0
β-Zearalenol	1.4	4.6	1.2	4.0
α-Zearalenol	1.0	3.3	0.9	3.0
Zearalanone	0.5	1.7	0.5	1.7
Zearalenone	0.2	0.7	0.2	0.7
Neosolaniol	0.1	0.3	0.1	0.3
15-Acetyldeoxynivalenol	0.8	2.6	0.8	2.6
3-Acetyldeoxynivalenol	0.8	2.6	0.8	2.6
Monoacetoxyscirpenol	0.1	0.3	0.1	0.3
Diacetoxyscirpenol	0.3	1.0	0.2	0.7
Aflatoxin M1	0.1	0.3	0.1	0.3
Aflatoxin B1	0.1	0.3	0.1	0.3
Aflatoxin B2	0.1	0.3	0.1	0.3
Aflatoxin G1	0.1	0.3	0.1	0.3
Aflatoxin G2	0.1	0.3	0.1	0.3
Fumonisin B1	0.5	1.7	0.5	1.7
Fumonisin B2	0.4	1.3	0.3	1.0
Fumonisin B3	0.5	1.7	0.5	1.7
T2 tetraol	5.4	17.8	4.5	14.9
T2 triol	0.3	1.0	0.2	0.7
Roquefortine C	0.2	0.7	0.1	0.3
Griseofulvin	0.1	0.3	0.1	0.3
T-2 toxin	0.1	0.3	0.1	0.3
HT-2 toxin	0.3	1.0	0.2	0.7
Ochratoxin A	0.1	0.3	0.1	0.3
Ochratoxin B	0.1	0.3	0.1	0.3
Mycophenolic acid	0.2	0.7	0.2	0.7
Mevinolin	0.1	0.3	0.1	0.3
